# Presence of Virulence Genes in *Enterococcus* Species Isolated from Meat Turkeys in Germany Does Not Correlate with Chicken Embryo Lethality

**DOI:** 10.1155/2019/6147695

**Published:** 2019-10-30

**Authors:** Julia Maasjost, Dörte Lüschow, Anne Kleine, Hafez M. Hafez, Kristin Mühldorfer

**Affiliations:** ^1^Institute of Poultry Diseases, Freie Universität Berlin, 14163 Berlin, Germany; ^2^Leibniz Institute for Zoo and Wildlife Research, 10315 Berlin, Germany

## Abstract

Virulence-associated traits have frequently been studied in enterococci and are considered to contribute towards the pathogenicity of infections. In the present study, *Enterococcus* isolates were collected during diagnostic investigations from meat turkeys in Germany. Twenty-eight isolates of three different *Enterococcus* species were analyzed for five selected putative virulence traits to understand their potential role in the pathogenicity using the chicken embryo lethality assay. Ten *E. faecalis*, ten *E. faecium*, and eight *E. gallinarum* isolates were examined for the presence of common virulence genes and their phenotypic expression, namely, the cytolysin operon, five individual *cyl* genes (*cylL*_*L*_, *cylL*_*S*_, *cylM*, *cylB*, and *cylA*), gelatinase (*gelE*), hyaluronidase (*hyl*_*Efm*_), aggregation substance (*asa1*), and enterococcal surface protein (*esp*). The *Enterococcus* isolates showed significant species-dependent differences in the presence of genotypic traits (*p* < 0.001 by Fisher's exact test; Cramer's *V* = 0.68). At least one gene and up to three virulence traits were found in *E. faecalis*, while six *E. faecium* isolates and one *E. gallinarum* isolate did not display any virulence-associated pheno- or genotype. More than half of the *Enterococcus* isolates (*n* = 15) harbored the *gelE* gene, but only *E. faecalis* (*n* = 10) expressed the gelatinase activity *in vitro*. The *hyl*_*Efm*_ gene was found in five *E. gallinarum* isolates only, while seven isolates showed the hyaluronidase activity in the phenotypic assay. In Cramer's *V* statistic, a moderate association was indicated for species (*V* ≤ 0.35) or genotype (*V* < 0.43) and the results from the embryo lethality assay, but the differences were not significant. All *E. gallinarum* isolates were less virulent with mortality rates ranging between 0 and 30%. Two *E. faecalis* isolates were highly virulent, harboring the whole *cyl*-operon as well as *gelE* and *asa1* genes. Likewise, one *E. faecium* isolate caused high embryo mortality but did not harbor any of the investigated virulence genes. For the first time, *Enterococcus* isolates of three different species collected from diseased turkeys were investigated for their virulence properties in comparison. The results differed markedly between the *Enterococcus* species, with *E. faecalis* harboring the majority of investigated genes and virulence traits. However, the genotype did not entirely correlate with the phenotype or the isolates' virulence potential and pathogenicity for chicken embryos.

## 1. Introduction

Enterococci are opportunistic bacterial pathogens that belong to the gastrointestinal flora of mammals and birds. *Enterococcus faecalis* and *E. cecorum* are responsible for the majority of enterococcal infections in poultry. *E. faecalis* is known to provoke amyloid arthropathy in layers [1] and the pulmonary hypertension syndrome in broilers [2]. This species is also a common bacterial cause of increased first-week mortality in chicks [3] and can cause hepatic granulomas in turkey poults [4]. Clinical infections with *E. cecorum* became more prevalent over the past years and are responsible for severe inflammatory lesions of bones, joints, and internal organs in various poultry species including turkeys [5]. Besides animals, enterococci are a common cause of nosocomial infections in humans, and therapeutic options are impaired by multiresistant strains [6, 7]. The role of livestock in contributing to antimicrobial and multidrug resistance by antibiotic treatment of large numbers of animals raise public health concerns and lead to a particular interest in virulence characteristics of circulating *Enterococcus* strains.

Resistance genes as well as virulence-associated genes are located on plasmids or transposons and can be transferred between different *Enterococcus* species and to other bacteria [8]. Several putative virulence traits have been described in enterococci. Cytolysin is a bacterial toxin with hemolytic activity, encoded by the cytolysin operon consisting of eight genes (*cylR1*, *cylR2*, *cylL*_*L*_, *cylL*_*S*_, *cylM*, *cylB*, *cylA*, and *cylI*) [9]. Gelatinase, encoded by the chromosomal *gelE* gene, is an extracellular zinc endoprotease that enables enterococci to hydrolyze gelatin, collagen, and other small peptides [10]. Enterococcal colonization of host tissues is also facilitated by degradation of hyaluronic acids [11], encoded by the hyaluronidase gene *hyl*_Efm_ [12], as well as by different adhesins. Aggregation substance, encoded by *asa1* [13], is a group of surface proteins that promotes bacterial adherence to renal tubular cells [14] and internalization by intestinal cells [15], while the enterococcal surface protein, encoded by *esp*, is associated with bacterial biofilm formation [16].

Animal infection experiments showed that some of these virulence traits may increase the pathogenicity of *Enterococcus* strains. In a rabbit model of *E. faecalis* endocarditis, mortality increased significantly in animals infected with bacterial strains that exhibited aggregation substance and cytolysin [17]. Another study indicated that strains with gelatinase activity seem to be more virulent in mice suffering from peritonitis than gelatinase-defective strains [18]. In humans, however, a study showed no association in enterococcal bacteremia cases between 14-day mortality and the presence of gelatinase, hemolysin, and the *esp* gene [19].

The presence of putative virulence genes and their expression *in vitro* do not allow definite conclusions about the virulence potential of bacterial strains under natural conditions [20, 21]. An alternative way to determine the virulence of a microorganism is the embryo lethality assay, which allows correlations with genotypic and phenotypic characteristics. Wooley et al. [22] used this laboratory-based assay as an alternative to chicken challenge models for differentiation of virulent and avirulent *E. coli* strains. A subsequent study of Gibbs et al. [23] aimed to determine whether different virulence traits of *E. coli*, isolated from healthy broilers and from broilers with colibacillosis, were suitable for the prediction of chicken embryo lethality results. Some traits correlated significantly with high embryo lethality; however, this correlation was not 100% based on a single trait. Sturzenhecker [24] used the embryo lethality assay to compare the presence of virulence traits with embryo test results of *Campylobacter jejuni* and *C. coli* isolates from poultry. She found no virulence-associated correlation for toxin- or flagella-producing isolates, whereas low-molecular-weight outer membrane proteins seemed to be characteristic for highly virulent isolates. Two recent studies by Borst et al. [25] and Jung et al. [26] investigated pathogenic and commensal *E. cecorum* isolates to compare the results from the chicken embryo lethality assay. The authors found significantly higher mortality in embryos infected with pathogenic isolates from poultry species and production systems where *E. cecorum* infections cause serious disease outbreaks.

The aim of the present investigation was to characterize 28 isolates of three different *Enterococcus* species collected from diseased turkeys in Germany based on their virulence properties and correlations between their genotype, phenotype, and embryo lethality.

## 2. Materials and Methods

The present study belonged to a doctoral project (Dr. med. vet.) that aimed to investigate the prevalence, antimicrobial resistance, and virulence of enterococci isolated in 2010 and 2011 from commercial poultry flocks in North Rhine-Westphalia and Lower Saxony, Germany [27, 28]. In the present investigation, twenty-eight isolates from meat turkeys belonging to three different *Enterococcus* species, namely, ten *E. faecalis*, ten *E*. *faecium*, and eight *E. gallinarum* isolates, were selected for phenotypic and genotypic characterization and determination of the embryo lethality index. The isolates were cultured during disease diagnostics from turkey poults with yolk sacculitis as well as from internal organs of subadult birds (Table 1) and were kindly provided by Poultry Clinics and Laboratory Dr. Pöppel (Delbrück, Germany). The initial bacterial identification was based on the multiplex PCR protocol from the study of Jackson et al. [29] with specific primers targeting the *sodA* gene [30] to differentiate between *E. faecalis*, *E. faecium*, and other *Enterococcus* species. Subsequently, a 16S rRNA gene analysis [31] was used for confirmation and to identify all isolates at the species level.

### 2.1. Detection of Virulence Genes

The following enterococcal virulence genes were investigated, namely, five cytolysin genes (*cylL*_*L*_, *cylL*_*S*_, *cylM*, *cylB*, and *cylA*), gelatinase (*gelE*), hyaluronidase (*hyl*_*Efm*_), aggregation substance (*asa1*), and enterococcal surface protein (*esp*). All target genes, primer sequences, and the corresponding references are listed in detail in Tables S1 and S2 in Supplementary Materials. Bacterial DNA extraction and multiplex PCR analyses were performed according to the protocols from the study of Vankerckhoven et al. [32]. Two additional PCR assays were conducted for separate detection of the *cylL*_*S*_ gene and the *cyl*-operon according to Camargo et al. [33] and Gaspar et al. [34], respectively. *Enterococcus faecalis* MMH594 (kindly provided by M. Gilmore, *Enterococcus* II initiative, Broad Institute (broadinstitute.org)) was used as a positive control in different PCR assays. Gene-specific products were confirmed by single PCRs and Sanger sequencing at LGC Genomics GmbH, Berlin, Germany.

### 2.2. Phenotypic Expression of Virulence Traits

All isolates were tested for their hemolytic (cytolysin) activity and gelatinase and hyaluronidase production *in vitro*. The expression of the *cytolysin activity* was tested on agar plates with 5% defibrinated horse blood (Oxoid, Wesel, Germany), incubated at 37°C for 24 to 48 hours. Only beta-hemolysis was assessed as positive reaction. The ability to *hydrolyze gelatin* was tested on nutrient gelatin plates (nutrient agar from Sifin Diagnostics, Berlin, Germany; nutrient gelatin from Oxoid) incubated at 37°C for 24 hours. After incubation, agar plates were stored at 4°C for 12 hours. Gelatin hydrolysis was indicated by clear zones around bacterial colonies [35]. *Hyaluronidase production* was tested on 7% Columbia sheep blood agar (Oxoid) incubated at 37°C for 24 to 48 hours using the *Streptococcus equi* decapsulation test [36]. *Staphylococcus aureus* ATCC 25923 was used as a positive control for phenotypic tests.

### 2.3. Embryo Lethality Assay

Specific pathogen-free (SPF) hatching eggs from VALO BioMedia GmbH (Osterholz-Scharmbeck, Germany) were used for the experiments. The chicken embryo lethality assay was conducted in 2014 as described by Maasjost [28], following established protocols for the inoculation of the allantoic cavity of ten-day-old chicken embryos [22, 37]. Bacterial growth curve experiments were performed in advance for the three *Enterococcus* species (strains K923/96, K808/97, and ATCC 49573) to adjust the inoculum by standardized optical density (OD) measurements as described by Maasjost [28]. Compared to Rudolph's [37] experiments, an inoculum between 250 and 500 colony-forming units (cfu) per egg was aimed and verified by viable bacterial cell counts. Twenty embryos were infected per isolate.

Ten embryos served as a negative control in each experiment and were inoculated with 200 *μ*l sterile PBS instead to evaluate the potential influence of the inoculation. In four infection experiments, uninoculated eggs at the same stage of incubation were placed into the same egg incubator to control for potential independent errors such as egg quality and environmental conditions.

Eggs were incubated at 37.7°C and 60–70% relative humidity and candled daily for seven days post infection (p.i.) to detect dead embryos. The death of embryos was defined as loss of blood vessels and absence of spontaneous movement [38]. Seven days p.i., all surviving embryos were sacrificed at −20°C for two hours. Dead and killed embryos were randomly examined for gross pathology and enterococcal growth. Samples from the yolk sac and, starting on day three, from the liver were plated on 7% Columbia sheep blood agar (Oxoid), incubated at 37°C for 24 hours, and evaluated.

Two well-characterized control strains, *E. faecalis* K923/96 (highly virulent) and *E. faecium* K808/97 (less virulent) as described by Rudolph [37], were kindly provided by Lohmann Tierzucht GmbH (Cuxhaven, Germany) and used as positive controls together with the reference strain *E. gallinarum* ATCC 49573 (isolated from the chicken intestine).

The embryo mortality rate (EMR) [22] and the embryo survival index (ESI) [37] were determined and used for virulence classifications (Table 2). The EMR was calculated for 20 infected embryos per isolate as the percentage of embryonic death after seven days. The ESI was determined adding the surviving embryos from 20 inoculated eggs from the first to the seventh day p.i. reaching a maximum value of 140 (20 embryos × 7 days). The classification according to the ESI was not always applied for the control strains because absolute numbers of embryos differed partly because of variable viability of the SPF eggs.

### 2.4. Statistical Analysis

The data analysis was performed in IBM SPSS Statistics version 25 by using descriptive statistics for nominal data. Fisher's exact test for small sample sizes was applied to assess significant differences (*p* < 0.05) in combination with Cramer's *V* correlation coefficient to estimate the strength of the association (0 = no association and 1 = perfect association). The categorical variables were as follows: *Enterococcus* species (*E. faecalis*, *E. faecium*, or *E. gallinarum*), presence of virulence traits by genotype (none, one, two, or more traits), age of the turkeys (poults or subadults), EMR (less, moderately, or highly virulent), and ESI (less, moderately, or highly virulent) corresponding to the contents in Table 1.

### 2.5. Ethical Statement

All experiments were performed in compliance with the animal protection laws of Germany. Experiments utilizing chicken embryos were terminated on day 17 of incubation that means four days prior to hatching [38].

## 3. Results

### 3.1. Detection of Virulence Genes

Table 1 lists the virulence genes detected in the 28 *Enterococcus* isolates. The PCR amplicons had the expected size in the gel electrophoreses and were confirmed by Sanger sequencing of short fragments and BLAST analyses (results not shown).

*gelE* was the most common gene detected in 15 out of 28 isolates, followed by *asa1* (12/28). Between one and up to five different *cyl* genes were found in nine isolates, while the *cyl*-operon was confirmed only in four *E. faecalis* isolates and the control strain K923/96 too. Predominant combinations were *cyl*-operon, *cylL*_*L*_*L*_*S*_*MBA*, *gelE*, *asa1* (*n* = 3; K923/96) and *cylAL*_*L*_*L*_*S*_*M*, *gelE*, *asa1* (*n* = 2). The *hyl*_*Efm*_ gene was exclusively found in *E. gallinarum* (*n* = 5). At least one of the virulence genes was found in *E. faecalis*, while six *E. faecium* isolates, two *E. gallinarum* isolates, and the corresponding control strains (K808/97 and ATCC 49573) were negative in the PCR analyses (Tables 1 and 3).

### 3.2. Phenotypic Expression of Virulence Traits

Ten *E. faecalis* isolates and the control strain K923/96 harbored the *gelE* gene and showed gelatinase activity. Four *E. faecium* isolates and one *E. gallinarum* isolate harbored the *gelE* gene too, but phenotypic tests were negative. Similarly, none of the *E. faecalis* or *E. faecium* isolates harboring *cyl* genes or the whole *cyl*-operon showed hemolytic activity (Table 1). Seven *E. gallinarum* isolates and the strain ATCC 49573 showed hyaluronidase activity. Five of the isolates and ATCC 49573 harbored the *hyl*_*Efm*_ gene; one isolate had only *gelE*, and one had none of the tested genes. Six *E. gallinarum* isolates and the strain ATCC 49573 were beta-hemolytic too, while none of the *cyl* genes or the *cyl*-operon was detected.

### 3.3. Embryo Lethality Assay

Concentrations of the inocula ranged between 136 and 856 cfu per egg for the 28 *Enterococcus* isolates under study with a mean of 431 cfu (Table 1). Inocula from the three control strains were within this range except for K923/96 that reached 10^3^ cfu in one experiment (Table 3). Dead embryos showed ecchymotic hemorrhages and subcutaneous edema characteristic of sepsis. Bacteriological investigations recovered *Enterococcus* in pure bacterial cultures from all infected dead and killed embryos tested. No embryo mortality was observed for negative controls during the experiments.

The observed EMR of the isolates (vs. the control strain) at day 7 p.i. ranged from 10 to 95% (80–100%) for *E. faecalis* (highly virulent K923/96), 0 to 70% (10–53%) for *E. faecium* (less virulent K808/97), and 0 to 30% (15–60%) for *E. gallinarum* (unclassified ATCC 49573). More than half of the isolates (*n* = 18) were found to be less virulent in both classification schemes, including five *E. faecalis*, five *E. faecium*, and all *E. gallinarum* isolates. Three isolates, two *E. faecalis*, and one *E. faecium* isolates were classified as highly virulent based on their ESI. Inconsistencies between both classification schemes were observed for two of them (*E. faecalis* and *E. faecium*), classified as moderately virulent by their EMR, and for one *E. faecium* that was classified as moderately virulent by its ESI and less virulent by its EMR (Table 1). The control strains showed discrepancies in their virulence classifications in the six repetitions (Table 3).

### 3.4. Statistical Analysis

The data from this study were analyzed to identify potential correlations between the *Enterococcus* species (“species”), the age of infected turkeys (“age”), and the genotypic presence of virulence traits (“genotype”) or results from the embryo lethality assay (“EMR” or “ESI”). Statistically significant, strong associations were found between the variables “species” (*p* < 0.001 by Fisher's exact test; Cramer's *V* = 0.68) or “age” (*p*=0.011 by Fisher's exact test; Cramer's *V* = 0.595) and “genotype.” Other tested relationships were not significant and revealed moderate or weak associations in Cramer's *V* statistic. Different analyses are summarized in Table 4; results from the chi-squared test were included for comparison.

## 4. Discussion

Putative virulence traits have frequently been studied in different enterococci of animal and human origin. They are considered to contribute to the pathogenicity of infections [39–41], but the underlying mechanisms often remain unclear. In the present study, 28 *Enterococcus* isolates from poults and subadult turkeys were investigated for five common virulence traits, namely, the cytolysin toxin, the lytic enzymes gelatinase and hyaluronidase, and the aggregation substance and enterococcal surface proteins. These traits have been selected because they seem to be more prevalent in the clinical course of *Enterococcus* isolates [17, 18, 40]. The aim was to understand genotype-phenotype correlations and their potential role in pathogenicity using the chicken embryo lethality assay.

The *Enterococcus* isolates revealed significant species-dependent differences in the presence of the genotypic traits (*p* < 0.001 by Fisher's exact test; Cramer's *V* = 0.68). The *E. faecalis* isolates harbored the majority of virulence genes investigated in this study. Six of ten *E. faecium* isolates and the corresponding control strain K808/97, however, did not show a virulence genotype or phenotype. Most *E. gallinarum* harbored the *hyl*_*Efm*_ gene only but showed beta-hemolytic and hyaluronidase activity. The latter two *Enterococcus* species are mainly concomitant bacteria in poultry diagnostics [42] but important nosocomial pathogens of humans and potential food contaminants [43, 44].

Phenotypic assays were performed for three out of five virulence traits investigating beta-hemolytic (cytolysin) and enzymatic (gelatinase and hyaluronidase) properties. Genotype-phenotype discrepancies were observed for some isolates and are described for the individual traits below. In general, the lack of phenotypic expression despite genetic evidence indicates the presence of variant (“loss-of-function”) or silent genes that can be activated under *in vivo* conditions [40, 45, 46]. Certain environmental factors such as temperature, ion concentration, or osmolality of the medium can downregulate the genetic expression and negatively affect the gene product [21, 40, 47]. Moreover, structural changes and divergent or newly acquired genes may account for hemolytic or enzymatic properties of isolates that are negative in PCR analyses [40].

The bacteriocin *cytolysin* has cytotoxic and hemolytic activity [48] and is one of the best-investigated virulence factors in enterococci [34, 49]. Between one and up to five different *cyl* genes were found in seven *E. faecalis* and two *E. faecium* isolates from turkeys, which is in good agreement with results from other studies in poultry [50–52]. Four *E. faecalis* isolates and the control strain K923/96 harbored the *cyl*-operon but did not show beta-hemolysis. In contrast, the majority of the *E. gallinarum* isolates and the strain ATCC 49573 were beta-hemolytic, but the corresponding *cyl* genes could not be confirmed.

The enzyme *gelatinase* catalyses the hydrolysis of proteins from the extracellular matrix [10]. Gelatinase activity may favor bacterial colonization and damage host cells [51, 53]. Although the *gelE* gene is very common in enterococci, its phenotypic expression can be impaired *in vitro* but seems to be predominant in clinical isolates from humans and animals [40]. In poultry species, concurrent gelatinase geno- and phenotypes have frequently been detected in *E. faecalis* from broilers and partridges, and more often than other virulence traits [51, 54–56]. These results are consistent with those from isolates of turkey origin under study. All *E. faecalis* isolates (including K923/96) harbored the *gelE* gene and showed gelatinase activity, while four *E. faecium* and one *E. gallinarum* isolates had silent genes only.

The enzyme *hyaluronidase* is responsible for the breakdown of hyaluronic acids, which facilitates bacterial colonization by decreasing the viscosity of the extracellular matrix [11]. In the present study, the *hyl*_*Efm*_ gene was detected neither in *E. faecalis* nor in *E. faecium*, but in five of eight *E. gallinarum* isolates. Interestingly, the strain ATCC 49573 and seven *E. gallinarum* isolates showed hyaluronidase activity, including those five isolates harboring the *hyl*_*Ffm*_ gene. One of the two remaining isolates was negative in all PCR assays, and the other harbored the *gelE* gene only. The knowledge about *E. gallinarum* and its pathogenic potential is still very limited, and virulence genes have only rarely been investigated so far [57, 58].

*Aggregation substance* and *enterococcal surface proteins* are likely to facilitate bacterial colonization and persistence in the host by promoting adhesion to surfaces and close bacteria-cell contacts [59]. In poultry, the *asa1* gene has been found in *E. faecalis* and in *E. faecium* isolates [56, 60, 61], whereas the esp gene could not be detected in several studies [51, 52, 62, 63]. In the present study, the *asa1* gene was present in eight *E. faecalis* and in four *E. faecium* isolates isolated from turkeys, while the *esp* gene could not be detected. The *E. gallinarum* isolates did not harbor the adhesin genes.

The 28 *Enterococcus* isolates (Table 1) and three control strains (Table 3) were investigated using the chicken embryo lethality assay to determine their virulence potential based on the embryo mortality rate (EMR) [22] and the embryo survival index (ESI) [37]. In both classifications, half of the investigated *E. faecalis* and *E. faecium* isolates and all eight *E. gallinarum* isolates were of low virulence (*n* = 18). The mean EMR was 55% in *E. faecalis* (*n* = 10), 31% in *E. faecium* (*n* = 10), and 14% in *E. gallinarum* (*n* = 8). A moderate association was indicated in Cramer's *V* statistic between species (*V* ≤ 0.35) or genotype (*V* < 0.43) and the results from the embryo lethality assay, but the differences were not significant (Table 4). These results seem surprising since the isolates were collected from clinically affected birds. Limitations in the availability of embryonated eggs from turkeys only allowed experimental infection of chicken embryos. Because *Enterococcus* isolates were collected from diseased turkeys, this restriction could affect the actual virulence potential and associated EMRs. Enterococci, however, are opportunistic pathogens that require certain conditions to cause disease and may also benefit from coinfections or underlying diseases [20, 64]. In a former study, Rudolph [37] tested 27 *Enterococcus* isolates that originated from joint swabs from brown egg-laying hens suffering from amyloid arthropathy, where *E. faecalis* is the primary pathogen [1]. Eighteen were identified as highly pathogenic, while the remaining isolates varied in their EMRs from 5 to 90% and were of low (*n* = 4) or moderate (*n* = 5) virulence. Another study investigated *E. cecorum* isolates from different sources by inoculation of embryonated SPF eggs from chicken and found animal-specific variations. Pathogenic isolates from poultry species and production systems, where disease outbreaks occur, had higher mean EMRs compared to isolates from birds like turkeys, where disease symptoms only appear sporadically [26].

Several factors (e.g., infection route, embryo quality, and age at inoculation) are known to influence embryo lethality results and impair comparability (summarized in [65]). Previous studies, however, were often not able to draw definite conclusions about whether the infective dose influences the response. These experiments included enterococci [37, 66] but also a variety of other bacteria (e.g., [38, 67, 68]). A very recent study by Blanco et al. [65] used *E. faecalis* K923/96 only and with different inoculum concentrations to define the median lethality dose in chicken embryos. The authors found strong positive correlations between the infective dose, the EMR, and the embryo survival time that should be considered for future experiments. The strain K923/96 originated from a chicken with amyloid arthropathy and was highly virulent in seven repetitions in the study by Rudolph [37]. This strain served as the control in the present experiments too, which were conducted in 2014 with equivalent inoculum concentrations. In two out of six repetitions, the strain K923/96 reached the upper classification limit for moderately virulent strains with an EMR of 80% (Table 3). Likewise, the second control strain K808/97 showed moderate virulence in two out of six repetitions with an increased EMR of about 50% but was defined as less virulent in Rudolph's experiments [37]. The inocula were with 248 and 352 cfu slightly higher than those in the other four repetitions but reached the intended range of 250 to 500 cfu in this study and were similar or less concentrated as those from the study of Rudolph [37]. The ATCC 49573 reference strain also varied in six repetitions between less and moderate in its virulence classifications. An inoculum-based tendency became apparent for this strain too, but not for K923/96 or the 28 isolates under study.

Differences between the two classification results were noticed for three *Enterococcus* isolates. They were classified more virulent by the ESI than by the EMR by taking the days p.i. into account when embryos died (Table 2). Isolates classified as highly virulent based on their ESI killed more than half (11–14) of 20 inoculated embryos within two days p.i. This initial high mortality is most probably caused by isolate-dependent abilities to grow and invade embryonic tissues, favoring early systemic infections [67]. Ten-day-old chicken embryos do not have a fully developed adaptive immune system to respond to invading pathogens [69].

The isolates from the present study originated from turkey poults with yolk sacculitis (*n* = 15) and from the internal organs of subadult birds (*n* = 13). Commercially raised poults experience different stressors during the first week of life and might be more prone to enterococcal infections compared to subadult birds [70]. Consequently, one would expect that *Enterococcus* infections in subadult turkeys are caused by virulent strains and by predominant species in poultry disease such as *E. faecalis*. The *Enterococcus* isolates from subadult birds belonged mainly to *E. faecalis* (*n* = 5) and *E. faecium* (*n* = 7). Significant age-dependent differences were identified in the presence of genotypic traits (*p*=0.011 by Fisher's exact test), while Cramer's *V* statistic also showed age-dependent, moderate to strong associations for the *Enterococcus* species (*V* = 0.46) and the virulence genotype (*V* = 0.595). Eight of 13 isolates from subadult turkeys harbored two or three of the investigated virulence traits, but the identified age differences did not correlate with the results from the embryo lethality assay.

A potential correlation between the isolates' virulence genotype and the pathogenicity for chicken embryos was further investigated independently of the age. Considering the theory of virulence traits conferring pathogenicity, one might expect that a higher virulence potential correlates with the presence of essential genes. Two highly virulent and a moderately virulent *E. faecalis* isolates indeed harbored the whole *cyl*-operon as well as *gelE* and *asa1* just as the control strain K923/96, which could enhance their virulence potential. Another *E. faecalis* isolate, however, had an identical genotype but was less virulent in the embryo lethality assay with comparable inoculum concentrations. This inconsistency became more evident with the highly virulent *E. faecium* isolate that did not show a corresponding geno- and phenotype (Table 1). Similar conclusions have been reached in virulence comparisons of pathogenic and commensal *E. cecorum* isolates assuming species-specific mechanisms [26].

## 5. Conclusion

Studies on enterococci often search for genes conferring virulence and antimicrobial resistance to identify potential threats to public health. Comparisons, however, reaching beyond and challenging the concept of virulence traits as intrinsic bacterial properties and determinants of pathogenicity [20] are rare but important for studying opportunistic or secondary pathogens. The three *Enterococcus* species under study belong to the intestinal microbiota of poultry but were isolated during disease diagnostics from clinically affected birds. As opportunists, their ability to cause disease in turkeys might rather be influenced by the host and its defense mechanisms than by isolate-specific virulence traits. Indeed, half of the *E. faecalis*, half of the *E. faecium*, and all *E. gallinarum* isolates under study were of low virulence in the chicken embryo lethality assay. The presence of virulence traits differed markedly between the three *Enterococcus* species, with *E. faecalis* harboring the majority of investigated genes. By comparing the results from this study, it became clear that the presence or absence of virulence genes or corresponding phenotypes did not entirely correlate with the isolates' virulence potential and pathogenicity for chicken embryos.

## Figures and Tables

**Table 1:** Inoculum concentrations (cfu), virulence genotype, phenotype and results from the chicken embryo lethality assay of 28 *Enterococcus* isolates from meat turkeys in Germany.

ID	Species	Age	Organ	cfu	Detected genes	Virulence traits by		†/*n*	7-day EMR		7-day ESI	
						genotype	phenotype					
2	*E. faecalis*	Subad	Heart	456	*cyl*-operon, *cylL*_*L*_*L*_*S*_*MBA*, *gelE*, *asa1*	CYL, GEL, ASA	GEL	8/20	40%	+	103	+
5	*E. faecalis*	Poult	Yolk sac	436	*cylL*_*S*_, *gelE*, *asa1*	GEL, ASA	GEL	13/20	65%	++	74	++
30	*E. faecalis*	Poult	Yolk sac	500	*cyl*-operon, *cylL*_*L*_*L*_*S*_*MBA*, *gelE*, *asa1*	CYL, GEL, ASA	GEL	19/20	95%	+++	39	+++
50	*E. faecalis*	Subad	Heart	852	*gelE*, *asa1*	GEL, ASA	GEL	7/20	35%	+	111	+
68	*E. faecalis*	Poult	Yolk sac	856	*gelE*	GEL	GEL	15/20	75%	++	53	++
87	*E. faecalis*	Poult	Yolk sac	428	*cylL*_*L*_ *L*_*S*_ *MA*, *gelE*, *asa1*	GEL, ASA	GEL	2/20	10%	+	127	+
137	*E. faecalis*	Poult	Yolk sac	588	*gelE*	GEL	GEL	6/20	30%	+	103	+
159	*E. faecalis*	Subad	Heart	484	*cylA*, *gelE*, *asa1*	GEL, ASA	GEL	7/20	35%	+	108	+
162	*E. faecalis*	Subad	Heart	684	*cyl*-operon, *cylL*_*L*_*L*_*S*_*MBA*, *gelE, asa1*	CYL, GEL, ASA	GEL	16/20	80%	++	50	+++
165	*E. faecalis*	Subad	Heart	772	*cyl*-operon, *cylL*_*L*_*L*_*S*_*MBA*, *gelE*, *asa1*	CYL, GEL, ASA	GEL	16/20	80%	++	52	++
6-I	*E. faecium*	Subad	Air sac	340	none	none	none	2/20	10%	+	127	+
44	*E. faecium*	Subad	Lung	304	none	none	none	0/20	0%	+	140	+
60	*E. faecium*	Subad	Air sac	648	*cylL*_*L*_ *L*_*S*_ *MA*, *gelE*, *asa1*	GEL, ASA	none	0/20	0%	+	140	+
69	*E. faecium*	Subad	Lung	212	*gelE*, *asa1*	GEL, ASA	none	1/20	5%	+	133	+
73	*E. faecium*	Subad	Air sac	304	*gelE*, *asa1*	GEL, ASA	none	12/20	60%	++	61	++
101	*E. faecium*	Poult	Yolk sac	376	*cylL*_*S*_ *A*, *gelE*, *asa1*	GEL, ASA	none	10/20	50%	++	76	++
151	*E. faecium*	Poult	Yolk sac	416	none	none	none	9/20	45%	++	82	++
153-II	*E. faecium*	Poult	Yolk sac	332	none	none	none	14/20	70%	++	48	+++
157-II	*E. faecium*	Subad	Lung	476	none	none	none	6/20	30%	+	103	+
160	*E. faecium*	Subad	Lung	176	none	none	none	7/20	35%	+	95	++
58	*E. gallinarum*	Poult	Yolk sac	288	*hyl*_*Efm*_	HYL	H, HYL	0/20	0%	+	140	+
77	*E. gallinarum*	Poult	Yolk sac	352	none	none	H, HYL	2/20	10%	+	135	+
79	*E. gallinarum*	Poult	Yolk sac	304	*gelE*	GEL	HYL	4/20	20%	+	122	+
94	*E. gallinarum*	Subad	Lung	288	none	none	none	2/20	10%	+	130	+
118	*E. gallinarum*	Poult	Yolk sac	280	*hyl*_*Efm*_	HYL	H, HYL	6/20	30%	+	122	+
127	*E. gallinarum*	Poult	Yolk sac	224	*hyl*_*Efm*_	HYL	H, HYL	3/20	15%	+	134	+
146	*E. gallinarum*	Poult	Yolk sac	404	*hyl*_*Efm*_	HYL	H, HYL	2/20	10%	+	135	+
156	*E. gallinarum*	Poult	Yolk sac	296	*hyl*_*Efm*_	HYL	H, HYL	4/20	20%	+	130	+

EMR, embryo mortality rate; ESI, embryo survival index; Subad, subadult; †/*n*, dead/tested embryos; CYL, cytolysin; H, beta-hemolysis; GEL, gelatinase; HYL, hyaluronidase; ASA, aggregation substance; virulence classification: +, less, ++, moderately, +++, highly virulent.

**Table 2:** Virulence classification according to the embryo mortality rate (EMR) [22] and embryo survival index (ESI) [37].

EMR (%)	ESI	Virulence
>80	0–50	Highly virulent
41–80	51–100	Moderately virulent
≤40	101–140	Less virulent

**Table 3:** Inoculum concentrations (cfu), virulence genotype, phenotype and results from the chicken embryo lethality assay (performed in six repetitions) of three control strains.

ID	Species	cfu	Genotype	Phenotype	†/*n*	7-day EMR		7-day ESI	
K923/96	*E. faecalis*	760	*cyl*-operon, *cylL*_*L*_*L*_*S*_*MBA*, *gelE*, *asa1*	GEL	20/20	100%	+++	15	+++
K923/96	*E. faecalis*	660			19/20	95%	+++	35	+++
K923/96	*E. faecalis*	376			18/20	90%	+++	45	+++
K923/96	*E. faecalis*	1000			18/20	90%	+++	40	+++
K923/96	*E. faecalis*	384			16/20	80%	++	52	++
K923/96	*E. faecalis*	548			8/10	80%	++	n.d.	n.d.

K808/97	*E. faecium*	352	None	None	10/20	50%	++	73	++
K808/97	*E. faecium*	224			6/20	30%	+	104	+
K808/97	*E. faecium*	144			4/20	20%	+	120	+
K808/97	*E. faecium*	152			2/20	10%	+	130	+
K808/97	*E. faecium*	248			8/15	53%	++	n.d.	n.d.
K808/97	*E. faecium*	168			1/10	10%	+	n.d.	n.d.

ATCC 49573	*E. gallinarum*	544	None	H, HYL	12/20	60%	++	72	++
ATCC 49573	*E. gallinarum*	324			11/20	55%	++	77	++
ATCC 49573	*E. gallinarum*	256			7/20	35%	+	99	++
ATCC 49573	*E. gallinarum*	136			6/20	30%	+	117	+
ATCC 49573	*E. gallinarum*	216			4/20	20%	+	124	+
ATCC 49573	*E. gallinarum*	232			3/20	15%	+	125	+

EMR, embryo mortality rate; ESI, embryo survival index; †/*n*, dead/tested embryos; H, beta-hemolysis; GEL, gelatinase; HYL, hyaluronidase; virulence classification: +, less, ++, moderately, +++, highly virulent; n.d., not determined because of differing absolute numbers of available viable embryonated SPF eggs.

**Table 4:** Results from the statistical analyses.

Tested variables	Fisher's exact test	Pearson chi-square test	Cramer's *V*^*∗*^
“Species” × “genotype”	*p* < 0.001	25.90, d*f* = 6, *p* < 0.001	0.680
“Age” × “genotype”	*p*=0.011	9.91, d*f* = 3, *p*=0.019	0.595
“Species” × “age”	*p*=0.052	5.99, d*f* = 2, *p*=0.050	0.462
“Species” × “EMR/ESI”	*p* ≥ 0.091	6.78/6.89, d*f* = 4, *p* ≥ 0.142	0.348/0.351
“Genotype” × “EMR/ESI”	*p* ≥ 0.202	9.26/10.14, d*f* = 6, *p* ≥ 0.119	0.407/0.426
“Age” × “EMR/ESI”	*p* ≥ 0.686	1.42/0.34, d*f* = 2, *p* ≥ 0.492	0.225/0.109

^*∗*^0 = no association, 1 = perfect association; EMR, embryo mortality rate; ESI, embryo survival index.

## Data Availability

Three partial 16S rRNA gene sequences (765 bp) from three isolates (IDs 2, 6-I, and 58), representing different *Enterococcus* species, have been submitted to the NCBI GenBank and are available under accession nos. MN387238–MN387240, respectively.

## Supplementary Materials

Supplementary Materials Table S1: details on multiplex PCR primers used for virulence gene detection. Table S2: details on primers used for individual PCRs and Sanger sequencing.
