# Supplementary material for: Presence of Virulence Genes in Enterococcus Species Isolated from Meat Turkeys in Germany Does Not Correlate with Chicken Embryo Lethality
Source: Biomed Res Int. 2019 Oct 30;2019:6147695. doi: 10.1155/2019/6147695 (PMC7012276; doi:10.1155/2019/6147695)
Supplement: Supplementary Materials — Table S1: details on multiplex PCR primers used for virulence gene detection. Table S2: details on primers used for individual PCRs and Sanger sequencing. [file 6147695.f1.pdf]

SUPPLEMENTARY MATERIAL

Presence of virulence genes in *Enterococcus* species isolated from meat turkeys in Germany does not correlate with chicken embryo lethality

Julia Maasjost[1], Dörte Lüschow[1], Anne Kleine[1], Hafez M. Hafez[1,§], Kristin Mühldorfer[1,2,§]

[1]Institute of Poultry Diseases, Freie Universität Berlin, 14163 Berlin, Germany
[§] contributed equally to this work

Correspondence should be addressed to Kristin Mühldorfer; muehldorfer@izw-berlin.de
[2]current affiliation: Leibniz Institute for Zoo and Wildlife Research, 10315 Berlin, Germany

Table S1: Details on multiplex PCR primers used for virulence gene detection

PCR target	Gene	Primer	Sequence (5' - 3')	Product	Reference
Cytolysin	*cylL$_L$*	CylLL1	GAT GGA GGG TAA GAA TTA TGG	253 bp	[1]
		CylLL2	GCT TCA CCT CAC TAA GTT TTA TAG		
	cylL$_S$	CylLS1	GAA GCA CAG TGC TAA ATA AGG	240 bp	[1]
		CylLS2	GTA TAA GAG GGC TAG TTT CAC		
	cylM	TE 13	CTG ATG GAA AGA AGA TAG TAT	742 bp	[2]
		TE 14	TGA GTT GGT CTG ATT ACA TTT		
	cylB	TE 15	ATT CCT ACC TAT GTT CTG TTA	843 bp	[2]
		TE 16	AAT AAA CTC TTC TTT TCC AAC		
	cylA	CYT_I	ACT CGG GGA TTG ATA GGC	688 bp	[3]
		CYT_IIb	GCT GCT AAA GCT GCG CTT		
Gelatinase	*gelE*	GEL 11	TAT GAC AAT GCT TTT TGG GAT	213 bp	[4]
		GEL 12	AGA TGC ACC CGA AAT AAT ATA		
Hyaluronidase	*hyl$_{Efm}$*	HYL n1	ACA GAA GAG CTG CAG GAA ATG	276 bp	[4]
		HYL n2	GAC TGA CGT CCA AGT TTC CAA		
Aggregation substance	*asa1*	ASA 11	GCA CGC TAT TAC GAA CTA TGA	375 bp	[4]
		ASA 12	TAA GAA AGA ACA TCA CCA CGA		
Enterococcal surface protein	*esp*	ESP 14F	AGA TTT CAT CTT TGA TTC TTG G	510 bp	[5]
		ESP 12R	AAT TGA TTC TTT AGC ATC TGG		

Table S2: Details on primers used for individual PCRs and Sanger sequencing

PCR target	Gene	Primer	Sequence (5' - 3')	Product	Reference
Cytolysin	*cylL$_L$*	CylLL1	GAT GGA GGG TAA GAA TTA TGG	253 bp	[1]
		CylLL2	GCT TCA CCT CAC TAA GTT TTA TAG		
	cylL$_S$	cylLS-F	AGA ACT TGT TGG TCC TTC	134 bp	[6]
		cylLS-R	GCT GAA AAT AAT GCA CCT AC		
	cylM	TE 13	CTG ATG GAA AGA AGA TAG TAT	742 bp	[2]
		TE 14	TGA GTT GGT CTG ATT ACA TTT		
	cylB	TE 15	ATT CCT ACC TAT GTT CTG TTA	843 bp	[2]
		TE 16	AAT AAA CTC TTC TTT TCC AAC		
	cylA	TE 17	TGG ATG ATA GTG ATA GGA AGT	688 bp	[2]
		CYT_IIb	GCT GCT AAA GCT GCG CTT		[3]
Gelatinase	*gelE*	TE 9	ACC CCG TAT CAT TGG TTT	213 bp	[4]
		GEL 12	AGA TGC ACC CGA AAT AAT ATA		
Hyaluronidase	*hyl$_{Efm}$*	hylEfm1	GAG TAG AGG AAT ATC TTA GC	661 bp	[7]
		hylEfm2	AGG CTC CAA TTC TGT		
Aggregation substance	*asa1*	ASA 11	GCA CGC TAT TAC GAA CTA TGA	375 bp	[4]
		TE 4	AAA CGG CAA GAC AAG TAA ATA		[2]

References

1. T. Semedo, M. Almeida Santos, P. Martins, M.F. Silva Lopes, J.J. Figueiredo Marques, R. Tenreiro, and M.T. Barreto Crespo, "Comparative study using type strains and clinical and food isolates to examine hemolytic activity and occurrence of the *cyl* operon in enterococci", *Journal of Clinical Microbiology*, vol. 41, pp. 2569-2576, 2003.
2. T.J. Eaton, and M.J. Gasson, "Molecular screening of *Enterococcus* virulence determinants and potential for genetic exchange between food and medical isolates", *Applied and Environmental Microbiology*, vol. 67, pp. 1628-1635, 2001.
3. T. Coque, J.E. Patterson, J.M. Steckelberg, and B.E. Murray, "Incidence of hemolysin, gelatinase, and aggregation substance among enterococci isolated from patients with endocarditis and other infections and from feces of hospitalized and community-based persons", *Journal of Infectious Disease*, vol. 171, pp. 1223-1229, 1995.
4. V. Vankerckhoven, T. Van Autgaerden, C. Vael, C. Lammens, S. Chapelle, R. Rossi, D. Jabes, and H. Goossens, "Development of a multiplex PCR for the detection of *asa1*, *gelE*, *cylA*, *esp*, and *hyl* genes in enterococci and survey for virulence determinants among European hospital isolates of *Enterococcus faecium*", *Journal of Clinical Microbiology*, vol. 42, pp. 4473-4479, 2004.
5. R.J. Willems, W. Homan, J. Top, M. van Santen-Verheuvel, D. Tribe, X. Manzioros, C. Gaillard, C.M. Vandenbroucke-Grauls, E.M. Mascini, E. van Kregten, J.D. van Embden, and M.J. Bonten, "Variant *esp* gene as a marker of a distinct genetic lineage of vancomycin-resistant *Enterococcus faecium* spreading in hospitals", *The Lancet*, vol. 357, pp. 853-855, 2001.
6. I.L. Camargo, M.S. Gilmore, and A.L. Darini, "Multilocus sequence typing and analysis of putative virulence factors in vancomycin-resistant and vancomycin-sensitive *Enterococcus faecium* isolates from Brazil", *Clinical Microbiology and Infection*, vol. 12, pp. 1123-1130, 2006.
7. I. Klare, C. Konstabel, S. Mueller-Bertling, G. Werner, B. Strommenger, C. Kettlitz, S. Borgmann, B. Schulte, D. Jonas, A. Serr, A.M. Fahr, U. Eigner, and W. Witte, "Spread of ampicillin/vancomycin-resistant *Enterococcus faecium* of the epidemic-virulent clonal complex-17 carrying the genes *esp* and *hyl* in German hospitals", *European Journal of Clinical Microbiology and Infectious Disease*, vol. 24, pp. 815-825, 2005.
